# When Zero Investment Does Not Mean Zero Financial Interest: Risks of Interpreting Ownership Data in Open Payments

**DOI:** 10.7759/cureus.115577

**Published:** 2026-09-01

**Authors:** Quynh Tran, Tucker Bettis, Logan Glosser, Felipe Maegawa, Snehal Patel

**Affiliations:** 1 Surgery, Emory University Hospital, Atlanta, USA; 2 Surgery, Emory University, Atlanta, USA; 3 General Surgery, Emory University Hospital, Atlanta, USA; 4 College of Law, Syracuse University, Syracuse, USA

**Keywords:** administrative data, conflicts of interest, financial disclosure, healthcare compliance, industry payments, open payments, physician ownership, sunshine act

## Abstract

Background

Open Payments is a national transparency program administered by the Centers for Medicare and Medicaid Services (CMS) that publicly reports financial relationships between reporting entities and covered recipients, including ownership and investment interests. The ownership file reports the amount invested during a single program year alongside the cumulative value of the interest held, so a report of zero investment accompanied by a positive cumulative value cannot establish when or how the interest was acquired. We measured how often this combination appears and how much investment history is recovered by reviewing earlier program years.

Materials and methods

We conducted a retrospective analysis of the CMS Open Payments ownership and investment interest detail files for program years 2014 through 2024, comprising 46,015 records and 43,097 physician-entity-years among 10,181 physicians. Records were aggregated to the physician-entity-year by summing annual investment and taking the maximum reported cumulative value. A physician-entity-year qualified when summed annual investment was zero, and maximum cumulative value was greater than zero. For each qualifying observation, we determined whether a positive investment had been reported for the same physician-entity relationship in an earlier program year, and we repeated the analysis at review depths of one to five prior years. Sensitivity analyses addressed linkage assumptions and a restricted definition requiring cumulative value above $1,000.

Results

Published records reporting zero annual investment with positive cumulative value increased from 312 of 5,459 (5.7%) in 2014 to 3,551 of 4,834 (73.5%) in 2024. The largest single-year change occurred between program years 2018 and 2019, coinciding with the boundary between archived and current publication versions; this discontinuity is unexplained, and the series should not be read as a continuous temporal trend. Among 19,798 qualifying physician-entity-years, earlier records reported a positive investment for the same relationship in 10,300 (52.0%), while 6,368 (32.2%) had no positive investment reported anywhere within the study window. Of the 10,300 prior investments identified through review of the complete available history, reviewing one prior year captured 3,446 (33.5%), three prior years captured 7,436 (72.2%), and five prior years captured 9,121 (88.6%). Across the principal linkage sensitivity analyses, the estimates ranged from 8,386 of 16,406 (51.1%) to 7,939 of 14,945 (53.1%).

Conclusions

Zero annual investment does not establish that an ownership interest was acquired without capital, and a prior reported investment does not establish the origin of a later reported interest. The public files contain no persistent identifier for an individual ownership interest, so an earlier investment and a later cumulative value within the same physician-entity relationship may describe different instruments. A single program year is a weak basis for characterizing a physician’s ownership interest. Longitudinal review of at least three to five prior program years is warranted before a reported ownership interest is acted upon. Only the effect of review depth was evaluated empirically; verification of physician and entity identity and corroboration with source documents proportionate to the consequences of the decision are proposed practices that have not been validated.

## Introduction

A hospital committee reviewing a physician’s public Open Payments record finds an ownership interest valued at $250,000 with zero investment reported during the program year. The record might be interpreted as indicating that the physician contributed no capital toward the interest. However, this conclusion is not supported by the field definitions because the investment may have occurred in an earlier year. Annual investment and cumulative value measure different time periods; therefore, a single program year can be misleading.

Open Payments is a national transparency program administered by Centers for Medicare and Medicaid Services (CMS) and established under the Physician Payments Sunshine Act. It requires applicable manufacturers and group purchasing organizations to report payments or other transfers of value to covered recipients and ownership or investment interests held by physicians or their immediate family members, and CMS publishes those data for public use [1-4]. The program is intended to promote transparency, not to determine whether a reported financial relationship is improper [4,5]. Certain physician-owned arrangements have received federal scrutiny, which demonstrates why ownership information may prompt institutional review without establishing that any particular interest is improper [6]. Previous research has documented substantial and highly concentrated financial relationships between physicians and industry, including ownership interests, but has generally analyzed the reported monetary values without examining their different temporal meanings [7-9]. A 2026 meeting abstract described the prevalence and distribution of ownership interests among oncology specialists from 2018 through 2024 [10]. The present study instead examines how the temporal structure of the reported fields affects longitudinal interpretation.

CMS defines the amount invested as the dollar amount invested during the program year, whereas the value of interest represents the cumulative value held as of the most recent feasible valuation date [5,11]. CMS does not prescribe a valuation method. The first field therefore measures annual investment and the second measures cumulative value, and a report of zero annual investment may coexist with an interest acquired in an earlier year.

This distinction matters wherever public records are consulted: conflict-of-interest screening for research and publication, credentialing and privileging, vendor and purchasing review, expert witness evaluation, litigation preparation, and external communication. Studies comparing self-disclosed financial relationships with Open Payments have found substantial differences, supporting the use of the database as a corroborating source while demonstrating that the two sources are not interchangeable [12]. The American Society for Health Care Risk Management (ASHRM) enterprise risk framework encourages healthcare organizations to identify, assess, and manage risks affecting operations, legal obligations, finances, strategy, and reputation [13]. Misinterpreting an ownership record may create exposure across several of these domains.

National studies examining how the different temporal definitions of these two fields affect longitudinal interpretation or quantifying how much investment history is recovered at different review depths are rare. The objectives of this study were (1) to measure how often zero annual investment coincides with positive cumulative value; (2) to determine how often earlier public records contain an investment for the same physician-entity relationship; (3) to quantify how the findings vary with the number of prior program years reviewed; and (4) to propose a framework for institutional review as an exploratory extension of these empirical objectives​​​​​​.

Objectives 1 through 3 were addressed empirically. Objective 4 was not formally validated and is presented as a proposed framework rather than as a finding of this study.

## Materials and methods

Data source and study period

We downloaded the CMS Open Payments ownership and investment interest detail files for program years 2014 through 2024 on July 10, 2026 [3]. Files for 2014 through 2018 were obtained from the archived datasets page and files for 2019 through 2024 from the current datasets page. Because CMS refreshes prior program years until they are archived, the file names, publication dates, download dates, and record counts for the files analyzed are shown in Table 1. Program year 2013 was excluded because it covered only the partial reporting period from August 1 through December 31, 2013. Program year 2025 was excluded from the primary analysis because only the initial publication was available at the time of data collection, although it was examined in a preliminary sensitivity analysis presented below. All published records from program years 2014 through 2024 were retained and aggregated as described below.

**Table 1:** Source file provenance Source: CMS Open Payments public-use ownership and investment interest detail files [3]. Program years 2014 through 2018 reflect the versions available when each year was archived. Program years 2019 through 2024 reflect the June 30, 2026, publication. Files for 2014 through 2018 were analyzed as downloaded CSV files. Files for 2019 through 2024 were analyzed from workbook copies created from the published CSV files without alteration of reported values, and record counts were verified against the published files. CMS: Centers for Medicare and Medicaid Services.

Program year	File identifier	Source page	Records, No.	Publication date	Download date
2014	OP_DTL_OWNRSHP_PGYR2014_P01212022	Archived	5,459	January 2022	July 10, 2026
2015	OP_DTL_OWNRSHP_PGYR2015_P01202023	Archived	5,087	January 2023	July 10, 2026
2016	OP_DTL_OWNRSHP_PGYR2016_P01182024	Archived	4,344	January 2024	July 10, 2026
2017	OP_DTL_OWNRSHP_PGYR2017_P01302025_01212025	Archived	3,323	January 2025	July 10, 2026
2018	OP_DTL_OWNRSHP_PGYR2018_P01302025_01212025	Archived	3,599	January 2025	July 10, 2026
2019	OP_DTL_OWNRSHP_PGYR2019_P06302026_06032026	Current	3,049	June 30, 2026	July 10, 2026
2020	OP_DTL_OWNRSHP_PGYR2020_P06302026_06032026	Current	3,555	June 30, 2026	July 10, 2026
2021	OP_DTL_OWNRSHP_PGYR2021_P06302026_06032026	Current	4,191	June 30, 2026	July 10, 2026
2022	OP_DTL_OWNRSHP_PGYR2022_P06302026_06032026	Current	4,148	June 30, 2026	July 10, 2026
2023	OP_DTL_OWNRSHP_PGYR2023_P06302026_06032026	Current	4,426	June 30, 2026	July 10, 2026
2024	OP_DTL_OWNRSHP_PGYR2024_P06302026_06032026	Current	4,834	June 30, 2026	July 10, 2026

Units, aggregation, and linkage

Analyses were conducted at three levels: the published record, the relationship between one physician and one reporting entity within a single program year, and the individual physician. We refer to the second level as the physician-entity-year. When a physician-entity-year contained more than one record, which occurred in 5.4% of physician-entity-years, we summed the annual investment amounts and used the highest reported cumulative value for that year. Annual investment was summed because separate records may report distinct contributions during the same year. Cumulative values were not summed because doing so could count overlapping descriptions of the same ownership relationship more than once. Because this aggregation represents an analytic choice rather than a verified feature of the data, we repeated the analysis among relationships containing only one record in every observed year. At the physician level, a physician was classified as having a qualifying observation if at least one attributed relationship qualified during that program year. Cohort derivation is shown in Table 2.

**Table 2:** Cohort derivation Source: This table was created by the authors from CMS Open Payments public-use ownership and investment interest detail files [3]. Program year 2013 (partial reporting period) and program year 2025 (initial publication only) were not included in the primary series. CMS: Centers for Medicare and Medicaid Services.

Step	No.	Change from preceding step
Published ownership records, program years 2014-2024	46,015	—
Aggregated to physician-entity-years	43,097	2,320 physician-entity-years contained more than one record; no record excluded
Distinct physician-entity relationships	11,292	—
Physician-entity-years with $0 summed annual investment	20,241	22,856 reported a positive amount
And maximum cumulative value greater than zero (primary cohort)	19,798	443 reported zero cumulative value
And cumulative value greater than $1,000 (restricted)	14,776	5,022 reported value of $1,000 or less

Physicians were linked across program years using the CMS physician profile identifier, and reporting entities were linked using the making payment identifier. Among 9,266 physicians with a National Provider Identifier (NPI) from 2015 through 2024, each physician profile identifier corresponded to one NPI, and each NPI corresponded to one physician profile identifier. Program year 2014 did not include an NPI field.

The files attributed 94.4% of reported interests to physicians and 5.6% to physicians’ immediate family members. We therefore describe the results as reported interests rather than as interests necessarily held personally by each physician. In 782 combinations of physician, reporting entity, and program year, the records included both physician-held and family-held interests. For the holder category sensitivity analysis, we assigned each combination to the category represented by most of its records and retained only relationships for which that category remained constant throughout all observed years.

The public file does not provide a persistent identifier for each ownership interest. Longitudinal linkage can therefore identify prior investment within the same physician-entity relationship, but it cannot establish that the investment corresponds to the specific interest reported in a later year.

Entity identifier screen

We used a rule-based screen to identify potential changes in entity identifiers. The screen was applied as the following ordered rules. First, we identified every reporting entity whose final observed program year preceded 2024 as a candidate predecessor and every entity whose first observed program year fell one or two program years after a predecessor's final year as a candidate successor. Second, both entities in a candidate pair were required to contribute at least 30 records across the study period; pairs involving smaller entities were not evaluated. Third, for each remaining pair, we computed the proportion of the successor's complete physician roster, identified by CMS physician profile identifier, that had previously appeared under the predecessor identifier. Fourth, for each retained pair, we also computed the Jaccard similarity of the two complete rosters. Fifth, a pair qualified when the proportion of the successor roster previously appearing under the predecessor exceeded 50%; Jaccard similarity was computed and reported for every candidate pair but was not itself a qualifying criterion. Sixth, a qualifying pair was treated as a single entity for longitudinal linkage only, and no record was excluded, and no monetary value was altered. One of the 2,054 candidate pairs qualified, so no rule was required to resolve a successor qualifying against more than one predecessor. The 50% threshold is a pragmatic choice intended to require continuity among a clear majority of the successor roster. It is not derived from any external standard, and neither the threshold nor the screen has been independently validated against corporate records or any other reference source. The complete implementation is included in the deposited analysis code.

Among 2,054 candidate pairs, one met the threshold. Of the physicians reported under SN Holdings, LLC, 98.4% had previously appeared under UroGPO LLC. The two complete rosters had a Jaccard similarity of 83.9%, meaning that 83.9% of physicians appearing under either identifier appeared under both. The next highest candidate overlap was 33.3%. The highest ranking candidate pairs and overlap measures are shown in Table 3.

**Table 3:** Entity identifier screen: highest ranking candidate pairs Source: This table was created by the authors from CMS Open Payments public-use ownership and investment interest detail files [3]. A total of 2,054 candidate pairs were evaluated, with both entities having at least 30 records and the successor first appearing one or two program years after the predecessor’s final year. Overlap is the proportion of the successor’s full physician roster previously appearing under the predecessor, and the specified analytic linkage threshold was 50%. One pair qualified. The complete ranked list of all 2,054 candidate pairs is included in the deposited analysis code and results dataset. This is an analytic linkage based on roster continuity and was not verified against external corporate records. CMS: Centers for Medicare and Medicaid Services.

Earlier identifier and final year	Later identifier and first year	Shared physicians, No.	% of successor roster	Roster Jaccard, %
UroGPO LLC (2020)	SN Holdings, LLC (2021)	239	98.4	83.9
OsteoReady LLC (2019)	Berman Instruments LLC (2021)	3	33.3	15.0
Paradigm Spine, LLC (2018)	Relievant Medsystems, Inc. (2019)	2	25.0	2.6
United Rheumatology (2017)	American Rheumatology Network (2019)	1	16.7	0.8
Ablative Solutions, Inc. (2023)	Vantis Vascular, Inc. (2024)	10	15.9	9.4
RT Oncology Services Corp. (2016)	Cornerstone Specialty Network LLC (2018)	10	12.7	2.7

This finding represents an analytic linkage based only on physician roster continuity. We did not verify a corporate relationship between the entities and do not assert that one existed. After linkage, individual records were retained and aggregated using the rules described above, and monetary values were not altered. Record-level estimates were unchanged when the linkage was omitted, and annual entity counts were unaffected because the two identifiers did not appear during the same program year.

Outcome definition

For annual descriptions at the record level, a record qualified if it reported an amount invested of zero and a cumulative value of interest greater than zero. For the primary longitudinal analysis, records were aggregated as described above, and a physician-entity-year qualified if its total annual investment was zero and its maximum cumulative value was greater than zero.

Both monetary fields were populated in all 46,015 records. No blank value was converted to zero, so all zero values represent numeric values reported in the source files rather than values created during data import.

As some records reported nominal cumulative values, we conducted a sensitivity analysis requiring the cumulative value to exceed $1,000. This restriction was applied after inspection of the value distribution rather than prespecified. Inspection showed a concentration of records reporting a cumulative value of $1 or less (4,789 records, 10.4% of all records), the large majority of which reported exactly $1; $1,000 was selected as a round value comfortably above that cluster. It corresponds to no statutory, regulatory, or institutional standard and should not be interpreted as an established materiality threshold. As the threshold is post hoc, the primary cohort, the primary results, and the conclusions of this study all use the unrestricted definition, and the restricted figures are reported only to demonstrate that the principal finding is not produced by nominal value reporting. Results using thresholds of $100 and $10,000 are presented below alongside the primary annual estimates so that the sensitivity of the estimates to this choice is visible. All analyses describe the published records and the physician-entity relationships derived from the specified data releases, not every ownership relationship that may have existed.

Longitudinal analysis

For each physician-entity relationship, we ordered the available records by program year and determined whether a positive investment had been reported during any earlier observed year. Observations meeting the outcome definition were classified into four mutually exclusive groups: (1) relationships first observed in the qualifying year, (2) continuing relationships with a positive investment reported in an earlier year, (3) continuing relationships with a positive investment reported only in a later year, and (4) continuing relationships with no positive investment reported anywhere in the 2014 through 2024 study period. The final category means that no positive investment was observed within the available data window, not that an investment was never made.

Because institutions must decide how many program years to review, we repeated the analysis using the one, two, three, four, and five program years immediately preceding each qualifying observation. At each review depth, we calculated both the percentage of all qualifying physician-entity-years for which a prior investment was identified and the percentage of the prior investments identified through review of the complete available history that were captured.

Observations from earlier program years had fewer prior years available for review. We therefore repeated this analysis using only observations from 2019 through 2024, for which at least five prior program years were available within the study window; recently established relationships could still have had shorter observed histories. Results of this restricted analysis are presented below.

We conducted four sensitivity analyses to assess the longitudinal linkage assumptions. First, we restricted the analysis to relationships containing exactly one record in every observed year. Second, we restricted it to relationships for which the ownership holder category remained constant across all observed years, and repeated the analysis separately for physician-held and family-held interests. Third, we required the broad ownership instrument category derived from the free-text terms field to remain constant across all observed years; instrument categories were stock or shares, partnership or membership units, options or warrants, and other. Fourth, we restricted the analysis to program years 2019 through 2024, avoiding comparisons across the boundary between archived and current publication versions.

Ownership instruments were classified from the free-text terms of interest field, which was populated in every record. The field value was converted to lowercase; no other normalization was applied, and punctuation, whitespace, spelling, and word forms were left unchanged. No record was excluded on the basis of this field. Three keyword rules were then applied in the order shown, and the first rule matched determined the category; records matching no rule were assigned to a residual category. Order is material because entries such as “stock options” or “membership units of common stock” contain terms belonging to more than one category.

Rule 1, options or warrants: Assigned if the text contained “option” or “warrant,” including their plural forms.

Rule 2, partnership or membership units: Assigned if not already assigned and the text contained “partnership” or “membership,” or the whole word “unit,” “units,” “LLC,” or “LP.”

Rule 3, stock or shares: Assigned if not already assigned and the text contained a word beginning with “stock,” the whole word “share” or “shares,” or the strings “equity,” “common,” or “preferred.”

Rule 4, other: Assigned to every remaining record, including records whose terms field described an instrument outside the three categories above or records whose terms field was uninformative.

A physician-entity relationship was included in the instrument-constancy sensitivity analysis only if every observed year of that relationship resolved to the same category. The classification was used for that sensitivity analysis alone and is not offered as a validated characterization of any reported interest. The complete implementation is included in the deposited analysis code.

Analysis and ethics

We summarized cumulative value distributions and entity concentration. Entity concentration was measured using the Herfindahl-Hirschman Index (HHI), defined as the sum of the squared percentage shares of records contributed by each reporting entity; on this scale, values approach zero when records are dispersed across many similarly sized entities and reach 10,000 when one entity contributes all records [14]. We repeated the analysis after excluding the three entities contributing the most records to the qualifying cohort across the entire study period, excluding the same three entities from every program year. We also repeated the analysis after excluding records for which the CMS dispute status field indicated that the record was disputed. As the data consisted of all published records in the specified releases rather than a sample, we report descriptive statistics without inferential testing. Analyses were performed using Python 3.12.3 (Python Software Foundation, Wilmington, DE) with pandas 2.2.2 and NumPy 1.26.4.

This study analyzed publicly available, nonclinical administrative data published by CMS. It did not involve human participants, patient data, or protected health information, and it did not constitute human subjects research, so institutional review board review was not required.

## Results

Cohort and annual pattern

The files contained 46,015 records representing 43,097 physician-entity-years, 11,292 physician-entity relationships, and 10,181 physicians. At the record level, the percentage reporting zero annual investment and positive cumulative value increased from 5.7% in 2014 to 73.5% in 2024, as shown in Table 4. When cumulative value was required to exceed $1,000, the percentage increased from 5.3% to 48.7%.

**Table 4:** Published ownership records and records meeting the outcome definition by program year Source: This table was created by the authors from CMS Open Payments public-use ownership and investment interest detail files [3]. A record met the primary outcome definition if it reported an annual amount invested of zero and a cumulative value of interest greater than zero. Amount invested applies only to the program year, whereas value of interest is cumulative. Percentages use all published records for the corresponding program year as the denominator. The final column applies the restricted definition requiring a cumulative value greater than $1,000. Counts in the fourth column correspond to the unrestricted percentages in the fifth column. CMS: Centers for Medicare and Medicaid Services.

Program year	Records, No.	Physician-entity-years, No.	Records with zero annual investment and positive cumulative value, No.	Records with zero annual investment and positive cumulative value, %	Records with zero annual investment and cumulative value greater than $1,000, %
2014	5,459	5,157	312	5.7	5.3
2015	5,087	4,832	776	15.3	13.5
2016	4,344	4,076	1,166	26.8	25.5
2017	3,323	3,108	1,211	36.4	33.4
2018	3,599	3,404	1,884	52.3	34.0
2019	3,049	2,843	1,747	57.3	51.6
2020	3,555	3,330	2,360	66.4	45.6
2021	4,191	3,969	2,522	60.2	43.6
2022	4,148	3,909	2,929	70.6	48.6
2023	4,426	4,121	3,046	68.8	49.4
2024	4,834	4,348	3,551	73.5	48.7

The difference between the unrestricted and restricted estimates increased sharply in 2018. Across the study period, 4,789 records (10.4%) reported zero annual investment and a cumulative value of $1 or less; of these, 4,172 reported exactly $1, and 4,785 involved physician-held interests. Nominal value reporting was concentrated by entity and program year. In 2018, one reporting entity contributed 507 of the 542 records reporting a cumulative value of $1 or less, and the annual number of such records increased from five in 2016 to 888 in 2024. This pattern explains a substantial portion of the difference between the unrestricted and restricted estimates. Estimates obtained using cumulative-value thresholds of $100 and $10,000, together with the magnitude-restricted measure computed at each unit of analysis, are shown in Table 5.

**Table 5:** Threshold sensitivities and unit of analysis Source: This table was created by the authors from CMS Open Payments public-use ownership and investment interest detail files [3]. The first four columns vary the cumulative-value threshold at the record level. The final three columns give the magnitude-restricted measure, requiring cumulative value greater than $1,000, at each unit of analysis. Similar estimates across units indicate that the pattern is not confined to relationships containing multiple records. CMS: Centers for Medicare and Medicaid Services.

Program year	Value >$0, %	Value >$100, %	Value >$1,000, %	Value >$10,000, %	Record level, %	Physician-entity-year level, %	Physician level, %
2014	5.7	5.7	5.3	2.8	5.3	5.2	5.6
2015	15.3	14.3	13.5	6.1	13.5	12.7	14.1
2016	26.8	26.3	25.5	13.1	25.5	24.1	25.6
2017	36.4	35.4	33.4	23	33.4	32.9	36.0
2018	52.3	36.8	34.0	25.7	34.0	33.2	35.5
2019	57.3	55.8	51.6	37.2	51.6	51.6	54.7
2020	66.4	48.4	45.6	35.6	45.6	45.0	46.9
2021	60.2	44.5	43.6	33.2	43.6	42.1	43.1
2022	70.6	53.7	48.6	34.3	48.6	48.1	49.0
2023	68.8	50.0	49.4	38.8	49.4	49.5	50.7
2024	73.5	53.6	48.7	39.8	48.7	50.7	51.9

The largest change in the restricted series occurred between 2018 and 2019, when the percentage increased from 34.0% to 51.6%. This change coincided with the boundary between archived and current publication versions. We found no corresponding change in CMS documentation concerning file structure, reporting guidance, validation rules, or entity coverage, although an undocumented change in data processing cannot be excluded [15]. We therefore do not interpret the increase from 2014 through 2024 as a continuous temporal trend. Within the current publication version, the unrestricted percentage ranged from 57.3% to 73.5% during 2019 through 2024.

Robustness

Estimates were similar across analytic levels. In 2024, the restricted measure was 48.7% at the record level, 50.7% at the physician-entity-year level, and 51.9% at the physician level (Table 5), indicating that the finding was not driven by relationships containing multiple records. Reporting was concentrated, with the 10 largest entities contributing between 38.7% and 57.1% of annual records, and after excluding the three entities contributing the most records, the restricted estimate for 2024 increased to 61.5% because the largest contributors reported proportionally fewer records meeting the outcome definition; annual concentration measures are shown in Table 6.

**Table 6:** Reporting entity concentration and estimates excluding the three largest contributors Source: This table was created by the authors from CMS Open Payments public-use ownership and investment interest detail files [3]. The Herfindahl-Hirschman Index (HHI) was computed on entity shares of records and scaled to 10,000 [14]. Reporting was concentrated. The three entities excluded are those contributing the most records to the qualifying cohort across the entire study period, and the same three are excluded from every program year. The principal pattern persisted after their exclusion. CMS: Centers for Medicare and Medicaid Services.

Program year	Entities, No.	Largest entity, % of records	10 largest, % of records	HHI	Measure excluding top three, %
2014	276	21.5	50.4	626	3.5
2015	264	21.5	49.6	602	10.5
2016	231	24.7	53.1	753	17.2
2017	242	9.2	39.0	241	23.4
2018	239	14.1	46.6	395	29.9
2019	248	10.3	38.7	275	40.7
2020	234	17.1	49.0	491	45.7
2021	242	14.8	54.0	539	43.5
2022	228	16.2	55.1	605	51.0
2023	234	17.2	54.5	639	61.6
2024	229	18.1	57.1	685	61.5

Excluding the 15 records coded by CMS as disputed changed no annual estimate by more than 0.1 percentage point. This analysis, together with the missing-versus-zero audit, the profile identifier stability check, and the preliminary program year 2025 estimates, is shown in Table 7.

**Table 7:** Additional sensitivity analyses Source: This table was created by the authors from CMS Open Payments public-use ownership and investment interest detail files [3]. NPI: National Provider Identifier; CMS: Centers for Medicare and Medicaid Services.

Analysis	Result
Excluding disputed records (n = 15)	No annual estimate changed by more than 0.1 percentage point
Entity identifier linkage removed	Record-level estimates identical; annual entity counts identical because the two identifiers never co-occur in a program year
Missing-versus-zero audit	No blank values in either dollar field in any program year (46,015 records); zeroes are numeric values in the source files rather than blanks created during import
Profile identifier stability, 2015-2024	No profile identifier mapped to more than one NPI and no NPI mapped to more than one profile identifier (n = 9,266)
Publication vintage blocks	Record-count decline through 2019 and recovery thereafter each present within the 2014-2018 and 2019-2024 blocks
Program year 2025 (preliminary)	n = 2,646; magnitude-restricted measure 59.1%; zero annual investment 64.2%; all records carried change type NEW at first publication
2018-2019 discontinuity	Unexplained. No corresponding change in CMS file structure, reporting guidance, validation rules, or entity coverage was identified

Information added by longitudinal review

Across program years 2014 through 2024, 19,798 physician-entity-years met the longitudinal outcome definition after aggregation. Earlier Open Payments records reported a positive investment for the same physician-entity relationship in 10,300 observations, or 52.0% of the cohort. Among the remaining 9,498 observations, 2,446 (12.4% of the full cohort) represented relationships first observed during the year evaluated, 684 (3.5%) had a positive investment reported only in a later year, and 6,368 (32.2%) had no positive investment reported anywhere within the study window. The full classification is shown in Table 8. These classifications describe reporting within the available data and do not establish whether or when an investment actually occurred.

**Table 8:** Longitudinal classification of qualifying physician-entity-years, 2014 through 2024 Source: This table was created by the authors from CMS Open Payments public-use ownership and investment interest detail files [3]. Percentages use all 19,798 qualifying physician-entity-years as the denominator and may not sum to 100.0 because of rounding. The absence of a reported positive investment within the study period does not establish that an investment never occurred. A prior reported investment also does not establish that it corresponds to the specific ownership interest reported in a later year. CMS: Centers for Medicare and Medicaid Services.

Classification	Observations, No.	%
Relationship first observed in the year evaluated	2,446	12.4
Continuing relationship with a positive investment reported earlier	10,300	52.0
Continuing relationship with a positive investment reported only later	684	3.5
Continuing relationship with no positive investment reported within the study period	6,368	32.2
Total	19,798	100.0

Effect of review depth

The yield increased with the number of prior program years examined, as shown in Table 9. Reviewing one prior year identified a reported investment in 3,446 observations, representing 17.4% of the primary cohort and 33.5% of the 10,300 observations identified through review of the complete available history. Reviewing three prior years identified 7,436 observations, representing 37.6% of the cohort and 72.2% of those identified through complete review. Reviewing five prior years identified 9,121 observations, representing 46.1% of the cohort and 88.6% of those identified through complete review. Reviewing three prior years therefore captured nearly three-quarters of the prior investments identifiable within the complete available history, and reviewing five prior years captured nearly nine-tenths.

**Table 9:** Prior reported investment identified according to the number of prior program years reviewed Source: This table was created by the authors from CMS Open Payments public-use ownership and investment interest detail files [3]. The third column uses all 19,798 qualifying physician-entity-years as the denominator. The fourth column uses the 10,300 observations with a prior reported investment identified through review of the complete available history as the denominator. Review depth was measured backward from the program year being evaluated. When a relationship had fewer prior years available than the specified review depth, all available prior years were included, so the estimates reflect both the selected review depth and the amount of historical information available for each observation. CMS: Centers for Medicare and Medicaid Services.

Prior program years reviewed	Observations with prior reported investment, No.	% of entire primary cohort	% of findings identified through complete available review
1	3,446	17.4	33.5
2	5,843	29.5	56.7
3	7,436	37.6	72.2
4	8,423	42.5	81.8
5	9,121	46.1	88.6
All available	10,300	52.0	100.0

Restricting the analysis to observations from program years 2019 through 2024, for which at least five prior program years were available within the study window, produced lower capture percentages at each review depth, as shown in Table 10. One prior year captured 29.4% of the observations identified through complete review, three prior years captured 65.5%, and five prior years captured 85.1%. The percentages from the full cohort are therefore modestly optimistic because relationships with shorter observed histories reach their complete available history after reviewing fewer years.

**Table 10:** Review depth restricted to program years 2019 through 2024 Source: This table was created by the authors from CMS Open Payments public-use ownership and investment interest detail files [3]. The analysis was restricted to the 14,945 qualifying physician-entity-years occurring in program years 2019 through 2024, each of which had at least five prior program years available in the study window. Complete review identified prior investment for 7,939 of these (53.1%), which is the denominator for the final column. Capture percentages are lower at every depth than the corresponding values in Table 9, indicating that the full-cohort estimates are modestly optimistic because relationships with short observed histories reach complete review at shallow depths. CMS: Centers for Medicare and Medicaid Services.

Prior program years reviewed	Prior investment identified, No.	% of subset	% of findings identified through complete available review
1	2,331	15.6	29.4
2	4,048	27.1	51.0
3	5,198	34.8	65.5
4	6,062	40.6	76.4
5	6,760	45.2	85.1
All available	7,939	53.1	100.0

Sensitivity analyses

The percentage with a prior reported investment ranged from 51.1% to 53.1% in analyses restricted to relationships with one record per year, a constant ownership holder category, physician-held interests, or program years within the current publication version, as shown in Table 11. Requiring the ownership instrument category to remain constant across all observed years produced an estimate of 47.2%. Among the 683 observations involving interests held by immediate family members with a constant holder category, 65.6% had a prior reported investment, although this estimate should be interpreted cautiously because of the smaller denominator.

**Table 11:** Sensitivity analyses of the prior reported investment finding Source: This table was created by the authors from CMS Open Payments public-use ownership and investment interest detail files [3]. The first row presents the primary analysis. Each subsequent row presents a separate sensitivity analysis and uses the number of qualifying observations shown in that row as its denominator. The final row applies the restricted outcome definition rather than a longitudinal linkage restriction. Ownership instrument categories were derived from the free-text terms field using a keyword rule. This classification was used only to test the effect of requiring a consistent broad instrument category across observed years and should not be interpreted as a validated characterization of the reported ownership interest. CMS: Centers for Medicare and Medicaid Services.

Analysis	Qualifying observations, No.	Observations with prior reported investment, No.	%
Primary analysis, all program years	19,798	10,300	52.0
Relationships with one record in every observed year	16,406	8,386	51.1
Holder category constant across the relationship	19,380	9,993	51.6
Physician-held interests only, holder constant	18,697	9,545	51.1
Immediate-family-held interests only, holder constant	683	448	65.6
Instrument category constant across the relationship	16,299	7,687	47.2
Program years 2019-2024 only	14,945	7,939	53.1
Cumulative value greater than $1,000	14,776	9,634	65.2

When cumulative value was required to exceed $1,000, a prior reported investment was identified in 65.2% of 14,776 qualifying observations. The full classification under that restricted definition is shown in Table 12.

**Table 12:** Longitudinal classification at the $1,000 cumulative-value threshold Source: This table was created by the authors from CMS Open Payments public-use ownership and investment interest detail files [3]. This table repeats Table 8 under the magnitude-restricted definition requiring a cumulative value greater than $1,000. The 21.0% in the final row applies to this restricted cohort only; the corresponding value for the primary cohort is 32.2%. CMS: Centers for Medicare and Medicaid Services.

Classification	Physician-entity-years, No.	%
First observed in year evaluated	1,388	9.4
Continuing; prior positive investment reported	9,634	65.2
Continuing; positive investment reported only later	655	4.4
Continuing; no positive investment reported in study window	3,099	21.0
Total	14,776	100.0

## Discussion

By 2024, nearly three quarters of published records reported zero annual investment with a positive cumulative value, and approximately half did so with a cumulative value greater than $1,000 under the post hoc restricted definition. This combination does not establish that an ownership interest was acquired without capital. It reflects the coexistence of two CMS fields that measure different periods: annual investment during the program year and cumulative value at the most recent feasible valuation date. The concern is therefore not the accuracy of either field but the limited information available from viewing a single program year.

The apparent increase across the study period should not be read as a continuous temporal trend. The largest single-year change in the restricted series occurred between program years 2018 and 2019, coinciding with the boundary between the archived files and the current publication version. We identified no corresponding change in CMS documentation concerning file structure, reporting guidance, validation rules, or entity coverage, and the public data do not allow us to determine whether the change reflects reporting behavior, the population of reporting entities, or differences in how archived and current files have been processed and refreshed. As the discontinuity is unexplained, comparisons that cross this boundary are uncertain, and estimates from 2019 through 2024 within a single publication version provide the more reliable characterization of current data. The longitudinal findings are subject to the same constraint, which is why the analysis was repeated within program years 2019 through 2024; the principal estimate was stable in that restricted analysis.

The principal practical finding is that earlier public records contained a positive investment for the same physician-entity relationship in 52.0% of qualifying observations. This provides historical context that is absent from a single program year, and it can be obtained from existing public files without purchasing or linking an external dataset, although staff time, identity verification, and careful interpretation remain necessary.

The analysis of review depth translates this finding into an operational parameter. In the full cohort, reviewing one prior year captured one third of the prior investments identified through review of the complete available history, three years captured nearly three quarters, and five years captured nearly nine tenths. In the analysis restricted to program years with at least five prior years available, the corresponding estimates were 29.4%, 65.5%, and 85.1%. The standard Open Payments search interface displays only the seven most recent program years, so examining earlier history requires downloading archived files separately [5]. The amount of historical context recovered therefore depends directly on the number of years reviewed. A three-year review may provide a feasible starting point, while decisions with greater potential consequences may warrant review of at least five years.

The inference supported by this analysis is limited, and that limitation is important. The public files do not provide a persistent identifier for each ownership interest. A positive investment reported in an earlier year and a cumulative value reported later may therefore represent different interests held by the same physician or family member in the same entity. Longitudinal review adds meaningful historical context, but it cannot establish the origin of a specific ownership interest. Estimates remained between 51.1% and 53.1% when analyses were restricted to relationships with one record per year, a constant ownership holder category, physician-held interests, or years within the current publication version. This consistency suggests that the principal descriptive finding is not materially altered by these linkage restrictions, but it does not establish that the earlier investment and the later cumulative value refer to the same interest.

Almost one third of qualifying observations had no positive investment reported anywhere within the available study period. For these observations, the public data cannot establish whether the interest predated Open Payments, arose before the entity began reporting, appeared previously under a different identifier, or was acquired through a mechanism that did not produce a reportable positive annual investment.

Implications for institutional review

Table 13 presents an author-developed taxonomy of potential interpretation errors, organized with reference to the ASHRM enterprise risk domains, together with a proposed review workflow. The present analysis directly supports longitudinal review, whereas the recommendations for identity verification and corroboration with source documents represent proposed practices that require further evaluation.

**Table 13:** Potential interpretation errors and proposed review workflow Source: This table was created by the authors. This author-developed taxonomy was informed by the ASHRM enterprise risk framework [13]. ASHRM has not endorsed the categories or the proposed workflow. Only the recommendation for longitudinal review is directly supported by the present analysis; the remaining actions reflect professional judgment and require validation. ASHRM: American Society for Health Care Risk Management; CMS: Centers for Medicare and Medicaid Services.

Potential error	What may go wrong	Proposed review action	Remaining uncertainty
Field interpretation	Annual investment is interpreted as the lifetime cost of acquiring the interest.	Confirm the CMS definitions of annual investment and cumulative value before drawing conclusions	Field meaning can be clarified, but the accuracy and completeness of the reported values remain uncertain
Temporal interpretation	Annual investment and cumulative value are treated as though they measure the same period.	Review a sufficient number of prior program years for the physician-entity relationship.	A prior investment may relate to a different ownership interest.
Identity linkage	The physician or entity is not linked consistently across program years or following an identifier change.	Verify physician profile identifiers and examine current and historical entity identifiers and names.	Identifier changes accompanied by substantial roster changes may remain undetected.
Valuation	Reported cumulative values are assumed to be comparable across entities or program years.	For consequential decisions, request documentation of the valuation date and method.	CMS does not prescribe a valuation method.
Governance	A consequential decision is based on an uncorroborated public record.	Request source documents concerning acquisition and valuation.	Source documents may be incomplete, unavailable, or disputed.
Reputational and strategic risk	A financial interest is characterized inaccurately in internal or public communications.	Route potentially adverse characterizations through established institutional communications and legal review.	The effect of the proposed workflow on decision quality has not been evaluated.

Review of a single program year may overstate concern by implying that an interest was acquired without capital, while dismissing the record may overlook a genuine conflict. The appropriate response is further review rather than accusation or disregard. Reviewers should distinguish among three levels of evidence: the record for a single program year, the longitudinal public history, and source documentation obtained from the physician or reporting entity. Source documents may provide information about the date and method of acquisition and the basis for valuation that is unavailable in the public files, although such documentation may also be incomplete or disputed.

CMS does not prescribe a valuation method, so reported cumulative values reflect estimates supplied by reporting entities and may not be comparable across entities or program years. The valuation date is also not published [5]. The free-text terms field may provide additional context, but it does not systematically report the acquisition date, method of acquisition, or basis for valuation. Entity identifiers may also change over time, so a search limited to a current identifier may fail to retrieve an investment reported under a different identifier in an earlier program year.

An unsupported inference may create institutional risk. Depending on the setting, it may lead to an incorrect conflict determination, an unnecessary recusal, an inconsistent vendor review, a weakened litigation or expert review position, or an internal or public statement that mischaracterizes a physician’s financial relationship. Potentially adverse external statements may warrant review through established institutional communications and legal processes. The scope of review should be proportionate to the potential consequences of the decision. A public record may be sufficient to initiate an inquiry but not to determine or characterize how an ownership interest was acquired.

Limitations

This study has several limitations. First, and most importantly, the public files do not provide a persistent identifier for each ownership interest, so earlier and later records within the same physician-entity relationship may describe different instruments. Longitudinal review identifies prior investment associated with the relationship but does not trace the origin of a specific interest. Second, the files do not permit identification of an individual family member, so an earlier physician-held interest and a later family-held interest within the same relationship cannot be distinguished with certainty; restricting the analysis to relationships with a constant ownership holder category reduced but did not eliminate this ambiguity, and 782 physician-entity-years contained records from both categories.

Third, the method used to aggregate multiple records within the same physician-entity-year represents an analytic choice rather than a verified feature of the data. Fourth, an interest may be acquired through a mechanism that produces no reportable positive annual investment, so the analysis cannot distinguish a newly acquired interest involving no reported contemporaneous investment from a continuing interest. Fifth, the available history was limited to program years 2014 through 2024, and the absence of a reported investment within this period does not establish that no investment occurred outside it. Sixth, archived files were preserved at different refresh dates, which may create artificial differences between program years. The discontinuity between 2018 and 2019 remains unexplained and coincides with the boundary between archived and current publication versions; it directly constrains interpretation of the apparent increase across the study period, which we therefore do not treat as a validated temporal trend.

Seventh, potential changes in entity identifiers were detected using physician roster overlap and were not validated against external corporate records; the 50% overlap threshold is a pragmatic analytic choice rather than a criterion derived from any external standard, and a different threshold could yield a different set of qualifying pairs. The screen identified only one qualifying pair and may not detect identifier changes accompanied by substantial changes in the physician roster. Eighth, cumulative values are reported by entities without a prescribed valuation method or a published valuation date. Finally, the files contain no information about clinical practice, referrals, purchasing, or utilization, and inclusion in Open Payments is not evidence of wrongdoing.

## Conclusions

Open Payments annual investment and cumulative ownership value must be interpreted according to the different time periods they describe. Review of the complete available history identified a prior reported investment for the same physician-entity relationship in about half of qualifying observations, and review of the three prior years captured nearly three-quarters of those findings, while review of five years captured nearly nine-tenths. Longitudinal review therefore provides important context that a single program year cannot supply, but it cannot establish the origin of a specific ownership interest. The public files contain no persistent identifier for an individual ownership interest, so an earlier reported investment and a later reported cumulative value within the same relationship may describe different instruments held by the same physician or family member.

A report of zero annual investment alongside a positive cumulative value should prompt further review rather than either an inference that the interest was acquired without capital or a decision to disregard the record. Review of sufficient depth, verification of physician and entity identity, and corroboration with source documents proportionate to the consequences of the decision may support the identification of genuine conflicts while reducing unsupported characterizations of continuing ownership interests. Among these, only the effect of review depth was evaluated empirically in the present study. Verification of identity, corroboration with source documents, and the remaining elements of the proposed review framework reflect professional judgment rather than validated outcomes, and their effects on decision quality have not been assessed.
